# Artificial Biomimetic Electrochemical Assemblies

**DOI:** 10.3390/bios12010044

**Published:** 2022-01-15

**Authors:** Tanja Zidarič, Matjaž Finšgar, Uroš Maver, Tina Maver

**Affiliations:** 1 Institute of Biomedical Sciences, Faculty of Medicine, University of Maribor, Taborska ulica 8, SI-2000 Maribor, Slovenia; tanja.zidaric@um.si (T.Z.); uros.maver@um.si (U.M.); 2 Faculty of Chemistry and Chemical Engineering, University of Maribor, Smetanova ulica 17, SI-2000 Maribor, Slovenia; matjaz.finsgar@um.si; 3 Department of Pharmacology, Faculty of Medicine, University of Maribor, Taborska ulica 8, SI-2000 Maribor, Slovenia

**Keywords:** MIP-based biosensors, MIP, molecularly imprinted polymer, biosensor, biomolecules, electroanalysis

## Abstract

Rapid, selective, and cost-effective detection and determination of clinically relevant biomolecule analytes for a better understanding of biological and physiological functions are becoming increasingly prominent. In this regard, biosensors represent a powerful tool to meet these requirements. Recent decades have seen biosensors gaining popularity due to their ability to design sensor platforms that are selective to determine target analytes. Naturally generated receptor units have a high affinity for their targets, which provides the selectivity of a device. However, such receptors are subject to instability under harsh environmental conditions and have consequently low durability. By applying principles of supramolecular chemistry, molecularly imprinted polymers (MIPs) can successfully replace natural receptors to circumvent these shortcomings. This review summarizes the recent achievements and analytical applications of electrosynthesized MIPs, in particular, for the detection of protein-based biomarkers. The scope of this review also includes the background behind electrochemical readouts and the origin of the gate effect in MIP-based biosensors.

## 1. Introduction

**Figure 1:**
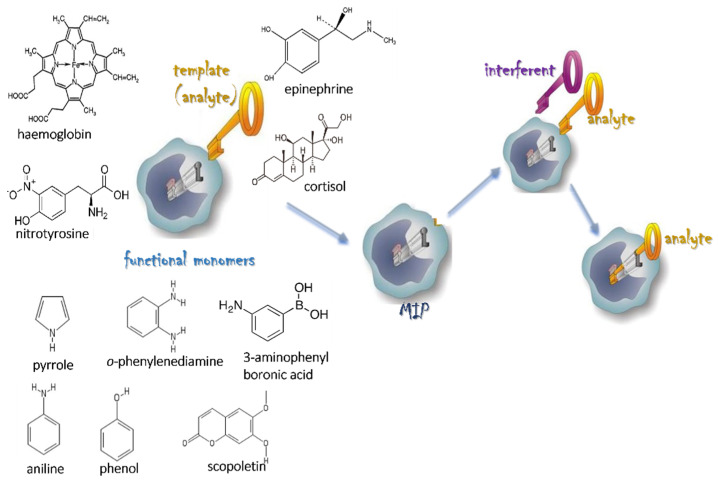
Theory behind the molecular imprinting: a pre-polymer solution containing a template (usually the analyte) and a polymerizable functional monomer is required. Under suitable conditions, many monomers surrounding the analyte transform into an artificial coating with the trapped analyte. After removing the template (analyte), specific cavities remain in the polymer matrix, and the size and chemical interactions complement the analyte molecule. During incubation with the analyte in the sample, the analyte binds specifically to the formed MIP (like the “lock and key” analogy). The figure shows the functional monomers most commonly used for electrosynthetic MIP in combination with some corresponding target analytes. The figure was prepared based on the information provided in the text of [4].

All living organisms know some form of naturally generated receptors that have an impressive ability to recognize target molecules specifically. This recognition presents the central event of almost all cellular interactions, such as enzymatic catalysis, nucleic acid hybridization, and antibody–antigen binding, among others [1,2]. Mimicking this ability to recognize and bind appropriate bioactive molecules in complex mixtures is an explicit fundamental goal of science and technology [3]. Especially in clinical settings, detecting target biomarkers is crucial for early disease detection, and subsequently for following disease progression. Most of these bioanalytical methods ensure their specificity by exploiting antibody–antigen interactions [4,5,6]. However, despite their specificity, they are subject to instability under harsh environmental conditions, on top of relatively expensive synthesis procedures and low durability [1,4,5]. Applying the principles of supramolecular chemistry, molecularly imprinted polymers (MIPs) can successfully replace natural receptors to circumvent these shortcomings [1,7]. These artificial biomimetic materials, often termed “smart” materials, can recognize target molecules based on their shape and size. Molecular imprinting is a template-based approach that leads to the formation of specific cavities in a 3D-polymer network (Figure 1). Subsequent template removal from the synthesized polymer matrix exposes cavities that reflect conformation and chemical functionalities of the target molecules [1,8].

As a result, MIPs have high affinity and selectivity similar to natural receptors when templates are prepared from polymers with molecular level structuring control. Putting the base selectivity aside, which might still be superior in nature, MIPs have several unique and practical features. For starters, they possess physicochemical stability superior to that of natural biomolecules and mostly a much longer shelf life. The recognition of MIPs is both mechanically and chemically stable under harsh conditions, such as a dry state, rendering them reusable in most cases [8,9,10,11]. The advantages of molecular imprinting can also be considered in terms of their fabrication, which further contributes to their attractiveness. These advantages include: (1) the simplicity of their production, making a specific recognition unit (i.e., a receptor) more readily available compared to relying on antibody production; (2) the versatility of possible templates, allowing MIPs to be used for recognition and rebinding of challenging analytes; and (3) the ease of adaptation to various biomedical applications, such as separation technologies, diagnostics, (bio)sensing, and drug delivery [4,8,9,10].

Electrochemical biosensing for clinically relevant biomolecules is a well-established technology that offers sensitive and specific detection accompanied by rapid response, user-friendly operation, portability, and real-time analysis [4,12]. It is well documented that early diagnosis is critical for improved patient outcomes, with rapid detection of clinical biomarkers playing a pivotal role [4]. Biosensors represent analytical devices that use a biological recognition element (i.e., a receptor) to obtain quantitative or semi-quantitative information without additional separation or processing steps. In this configuration, the biochemical receptor provides selectivity, while the transducer acts as a converter of a biological signal into a quantifiable electronic signal [5,13]. The bioreceptor moiety is the heart of any effective biosensor, as it defines high sensitivity, a low limit of detection (LOD), and good selectivity for the analyte of interest. An ideal biosensor should also ensure the overall quality and robustness of the obtained results [8,12,14]. In addition, the miniaturization of a biosensor is also considered a valuable feature for a practical application, especially with the increasing trend towards point-of-care devices [14]. Biosensors contain antibodies, enzymes, nucleic acids, or aptamers as the recognition unit that provides the desired selectivity [15]. However, the limitations of biosensors go hand in hand with the limits of their natural receptors. Therefore, great efforts have been made to search for new innovative materials capable of binding a target analyte with an affinity similar to natural receptors [8,15,16,17]. Polymer-based receptors are of particular interest as promising candidates, as alternatives or to complement natural recognition units due to their ability to be mass-produced, easy handling, low cost, improved durability, and potential to minimize batch-to-batch performance variation [8,14,16]. Among a plethora of polymer-based matrices for biosensing applications, MIPs are commonly used as artificial recognition moieties, particularly as “synthetic antibodies” referred to as “plastobodies”, to emphasize the analogy to specific binding by antibodies [2,8,9,15]. Molecular imprinting technology has been adopted for the electroanalytical determination of various antibiotics [18,19,20,21], herbicides [22,23], pesticides [24], drugs [25], and macromolecular compounds, from oligonucleotides [26,27] to carbohydrates [28,29,30], and proteins [4,15,17,31]. Unlike small molecular targets, proteins show “special” properties, such as their size, irreversible conformational changes, and secondary and tertiary structure. All mentioned have hindered the progress in protein imprinting for years [32].

Imprinting of materials has attracted the attention of many scientific groups, which is reflected in a vast range of different applications. This approach has been successfully used in separation processes as sorbents, chromatographic and electrophoretic separation, development of new magnetic or quantum dot materials, and clinical, food safety, and environmental analysis. In this context, a wealth of different synthetic strategies were presented to facilitate the design of efficient, imprinted materials for the application of interest. More detailed explanations of the synthesis, application, and limitations of MIPs can be found recent reviews [2,12,14,17,24,27,33,34,35,36,37,38,39,40,41,42,43,44,45,46,47,48].

In this review, the concepts of molecular imprinting technology for developing biomimetic electrochemical sensors are presented along with the current trends and challenges, particularly for biomarker recognition, focusing on electrosynthesized MIP (eMIP) biosensors. The associated electrochemical readout and the so-called “gate effect” phenomena are also discussed.

## 2. Molecular Imprinting Technology for Protein Recognition

The beginnings of molecular imprinting can be dated back to 1936 by Polyakov, who established the basic principle of forming a polymeric network around a small extractable template molecule [2,6,49]. According to the literature [1,4], the pioneering work was performed in 1949 by Dickey [50,51]. In their work, they have introduced some crucial fundaments of today’s molecular imprinting technology, such as identifying the target molecule as a “template” [4,50]. In the 1970s, Wulff et al. [52] and Kloatz et al. [53] further refined the basic concepts of MIP preparation by imprinting template molecules into organic polymers. Their work opened new horizons to create artificial binding sites (i.e., molecular cavities) in a precise manner [1]. In the 1980s, Mosbach et al. [54,55,56] first introduced a non-covalent imprinting approach, which is more common in current research [4]. The same group made a real breakthrough in 1993 [57] when they applied a radiolabeled molecular imprinted sorbent assay that mimicked antibody binding sites. This study demonstrated the potential of MIPs to replace antibodies in biosensor development [1,57].

**Figure 2:**
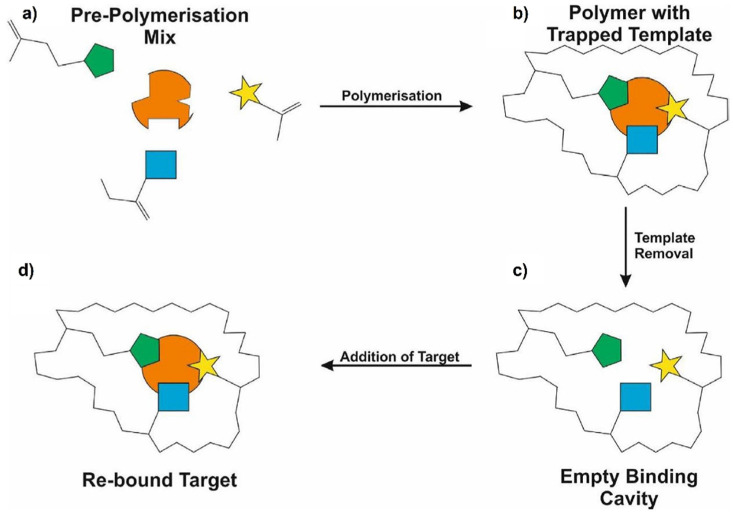
Schematical depiction of the molecular imprinting general idea: (**a**) a pre-polymerization mixture ensures that the non-covalent interactions stabilize between the template and functional monomers, followed by the polymerization; (**b**) the template is entrapped in the polymer matrix; (**c**) after template removal, a molecular cavity of complementary size, shape, and functionality to the target is created inside the polymer; (**d**) when the target is reintroduced, it will bind back inside the cavity [4].

As mentioned above, molecular imprinting involves the synthesis of highly cross-linked polymers that enable specifical molecular recognition [58]. Typically, the imprinting process (Figure 2) is performed in the presence of a target molecule (i.e., a template) around which the polymerization of a functional monomer occurs. It involves three steps: (1) pre-assembly of the functional monomer around the template molecule in the pre-polymerization solution by forming covalent bonds (pre-assembled approach) or by self-assembly through non-covalent bonds; (2) polymerization of the resulting complex, with the template molecule in the polymeric matrix by the presence of appropriate initiators (monomers, to which functional groups capable of cross-linking the polymeric structure are attached) and/or by physical stimulation (temperature, UV, applied current or potential); and (3) removal of the template from the synthesized polymeric network [1,4,5,17,31,58], resulting in the formation of molecular cavities. Since the first step is crucial for generating highly selective cavities, functional monomers are often modified with the attached functional groups capable of recognizing and binding the specific group of the template molecule used. This, in turn, enables the intermolecular interactions (hydrogen bonding, dipole–dipole and ionic interactions) between the template molecule and the functional groups in the polymer matrix, thereby controlling the molecular recognition phenomena [1,17,58]. However, in the case of conductive polymers, the monomers are usually used without the aid of cross-linkers, as they can be “overoxidized” to form functional groups that form intermolecular interactions more efficiently [5,17].

Depending on the intended application, the ideal functionality and sensitivity of the formed MIP in the development of biomimetic electrochemical sensors can be achieved through a variety of synthetic fabricating routes [4,5], which are listed in Table 1. In addition to the listed synthesis methods, MIP formation can also be achieved by UV polymerization, thermal polymerization, reversible addition-fragmentation chain transfer (RAFT) polymerization, and solid-phase synthesis [4].

In conventional bulk imprinting, polymerization of the monomer–template complex is initiated by either a photoinitiator or a thermal initiator. To obtain MIP particles from the synthesized bulk polymer, a bulk rigid polymer is firstly mechanically ground, followed by the solid-phase template extraction through prolonged washing with protic solvents [2,6]. One difficulty of classical bulk imprinting methods is the frequent entrapment of template molecules in the polymer layer, which hinders the removal and rebinding of a target/template molecule [2,32].

### 2.1. Protein Imprinting

Good knowledge of the intermolecular interactions between the binding sites of a target analyte and the recognition sites of the imprinted cavities that facilitate molecular recognition is crucial for an efficient MIP design. Different approaches of traditional bulk polymerization are efficient for low-molecular-weight templates (200–1200 Da), although they are generally unsuccessful for structurally complex macromolecules, such as proteins. Although there are many suitable targets for molecular imprinting, from pharmaceutical and chemical compounds to biological macromolecules, the complexity of proteins complicates the imprinting process. [2,31,32,59,60]. As mentioned above, the reason for this shortcoming mainly lies in the peculiarities of protein templates. Due to their fragile nature, they can irreversibly change their conformation during the polymerization process [32,59,61]. Consequently, the deformed cavities in a polymer matrix do not match the native conformation of the target. The (often) large size of the proteins increases the possibility of irreversible entrapment in the polymeric network, leading to limited accessibility of the imprinted binding sites [2,31,32]. Since proteins are flexible macromolecules that respond to environmental conditions and external stimulators (temperature, ion concentration, pH), this often leads to changes in their structure and functionality. Among many other parameters, electrostatic interaction is one of the most important factors for proteins’ adsorption, conformation, and stability on various surfaces and interfaces [62]. Subsequently, the large number of potential interactions on the protein’s surface may cause cross-reactivity of the imprinted polymers and non-specific adsorption to a bulk polymer. Therefore, the range of imprinted protein templates is limited to proteins with good conformational stability and distinct physicochemical properties, including a high isoelectric point and glycosylation. The isoelectric point of proteins can also determine the selectivity of a protein-imprinted polymer by facilitating the formation of strong and/or specific intermolecular interactions [32,63], i.e., electrostatic interactions between positively charged proteins (lysozyme [64], avidin [65]) and negatively charged polymers. In the case of lysozyme, most of the cationic residues are arginine, which can provide more non-covalent intermolecular interactions. Considering polymers with protein imprinting, polymers with hydrogen bond acceptors and anionic functional groups are expected to show a tendency for lysozyme. Another example is glycosylation, which is often used to achieve selective recognition of glycoproteins in boronic acid-containing polymers [63]. In this case, intermolecular interactions are formed between glycan moieties and aminophenyl boronic acid (APBA) monomers [32,66]. The resulting polymer, designed to bind the diols of a glycoprotein, would, in turn, preferentially bind the more glycosylated protein [63,67].

For nearly two decades, various approaches have been investigated to overcome the drawbacks of protein imprinting. First, to avoid the denaturation of a protein template, water-soluble functional monomers and initiators are chosen. However, there are concerns that the water molecule affects the hydrogen bonding and dipole–dipole interactions between the monomers and the template [32]. Initial attempts to use different strategies of protein-MIPs took the form of lightly cross-linked hydrogels with large pores, similar to those used in gel electrophoresis [68,69]. Unfortunately, the insufficient chain flexibility of such a polymer matrix has not met the requirements for the quality and stability of the prints in the initial state [32,70].

More significant popularity has been gained by the idea of “epitope imprinting”, in which a small, representative polypeptide sequence is used as a template instead of a whole protein molecule [32,71,72]. This approach is superior to the other conventional strategies for several reasons, including the relative ease of template removal, the preparation of uniform binding sites, and lower synthesis costs. In addition, polypeptide templates are far less exposed to the effects of their environment and do not exhibit secondary or tertiary structures [3,60]. The disadvantage of this approach can be associated with antibody production, namely the complex procedure of finding suitable linear peptides that are identical in sequence to one of the terminal peptide chains of the target protein. This requires detailed knowledge of the 3D structure of the protein in order to achieve a conformational arrangement of the epitope that is accessible to the antigen-binding region (paratope) of an antibody or synthetic recognition site [3,60,73].

A surface imprinting technique, whose main feature is an adjustment of the polymer layer thickness, has partially solved the constraints of macromolecular imprinting [2,31,32]. In some cases, proteins are even trapped by two-dimensional (2D) monolayers of suitable functional monomers anchored on the surface of a substrate [32,74].

Established synthesis methods from polymer chemistry have been constructively adapted for the preparation of MIP particles to maximize the binding capacity due to the high surface-to-volume ratio [2,31,32], including:

- preparation of micro- or nano-MIPs by precipitation or (mini or micro) emulsion polymerization leading to uniform spherical polymer particles under suitable conditions [3,75];
- generation of MIP nanoparticles (NPs) based on solid-phase synthesis mode [3,76];
- design of MIP-covered core-shell by core-shell grafting (formation of solid nanocore followed by grafting of imprinted shell), which can be post-functionalized with fluorescent, polyethylene glycol or anchor groups [3,77,78].

**Figure 3:**
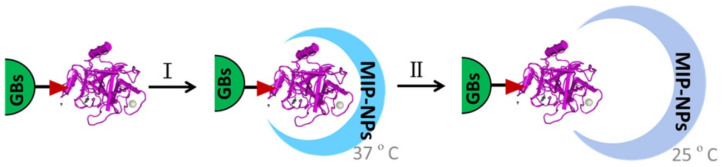
Schematic depiction of the solid-phase synthesis of MIP-NPs. I: polymerization; II: release of thermoresponsive MIP-NPs (temperature change from 37 °C to room temperature, 25 °C). Reprinted with permission from [76]. Copyright 2016 American Chemical Society.

Solid-phase synthesis is considered to be an advanced MIP manufacturing technique consisting of three main steps (Figure 3): (1) preparation of activated and silanized glass beads; (2) followed by immobilization of the template on silanized beads; and finally, (3) their polymerization and purification [3,79].

Silanized glass beads simultaneously play the role of a reactor and an affinity purification column [76]. The features of the solid-phase imprint include the possibility of reusing the template attached to the solid-phase and the orientation control over the polymer-template interactions. The latter contributes to a more homogeneous distribution of binding sites [2,3,80]. Such interfacial polymerization can generate ultrathin polymer films that only partially cover a protein template without completely enclosing it. In combination with thermoresponsive polymers, a thermo-controlled release of the template is possible, making MIPs virtually template-free, and eliminating the problem of template leaching or bleeding [3,76].

Microcontact printing and MIP nanosphere lithography, a former type for oriented imprinting, are promising approaches for protein imprinting that can circumvent the associated problems of solubility, conformational stability, and aggregation during polymerization [64,81]. Both methods offer the rapid preparation of MIPs using small amounts of template and monomer solution with the potential to prepare multiple samples simultaneously using the same polymerization approach [3,81,82]. A typical process begins with the immobilization or adsorption of a template onto the surface of a substrate (e.g., glass beads) to create the protein stamp, which is contacted with monomers in the following step. After a polymerization process, the substrate is removed, and an imprinted thin film with a stamp-like surface patterning is formed [2,3].

Coupling principles of electrochemistry with a molecular imprinting technique provides another efficient route for synthesizing MIPs, especially for developing MIP-based electrochemical sensors. Electropolymerization can be used to generate ultrathin MIP films by oxidation of an electroactive monomer directly on the surface of electrodes [59,71,83]. In this process, the electroactive monomers and a template are self-assembled via covalent or non-covalent interactions on the surface of the conductive substrate [59,83]. The electroactive monomers are polymerized on the substrate surface by applying a current or potential. At the same time, the template molecules are entrapped in the polymer film and form the binding sites in the polymer matrix. By simply adjusting the parameters during the electrooxidation of the monomers, a polymer film of the desired thickness can be fabricated. In addition, this is a quick and easy way to create an adherent film that cannot be easily peeled off the surface of the substrates. On the other hand, electropolymerization is limited to conductive surfaces. The integration of nanomaterials with a metallic character, such as graphene, carbon nanotubes (CNTs), metal and metal oxide NPs, can increase the binding capacity and electroactive surface for an effective signal generation [2,59,83,84].

### 2.2. Removal of Protein Template

Once the polymerization process is complete, the next step of template removal is needed to free the imprinted sites and enable the rebinding of target molecules. Large templates and the highly cross-linked polymer matrix of MIP have a joint negative effect on releasing a template molecule [3,85]. Unsuccessful template removal leads to so-called “template leakage” or “template bleeding”, which can contribute to false-positive signals in molecular target detection [86]. The crucial role of template removal is unique within affinity sensing and complicates the general applicability of MIPs [2,87]. In the absence of a universal method (each target molecule requires some specific conditions), various chemical and physical treatments (or combinations thereof) have been proposed to remove the protein template from the polymeric network, including changing the pH or ionic strength, elevated temperature, detergents, electrode potential, or ultrasound [2,88,89,90]. Proteolytic digestion is particularly favorable for template removal under mild conditions. However, it must be considered that protein fragments may be tightly bound to the MIP [91]. Therefore, compromises must be made between the efficiency of template removal and the integrity of the polymer [2].

### 2.3. Heterogeneity of Imprinted Sites

Conventional bulk imprinting, especially concerning protein imprinting, faces numerous challenges, including the high proportion of non-specific binding in the MIP. This heterogeneity of binding sites in bulk MIP is related to the random orientation of the template–monomer complex in a polymeric network, variations in the way the template is complexed, and even the presence of template–template interactions [3,92,93], as was observed in the dimerization of anaesthetic bupivacaine due to the formation of hydrogen bonds [93]. The relatively large surface area of MIPs compared with the number of high-affinity sites can lead to direct non-specific binding between the template and interfering molecules, which often hinders the suitability of MIPs in sensing applications [3].

To avoid the non-selectivity of the imprinted sites, it is necessary to select suitable functional monomers as the building block for the desired template. Strong interactions are required to form a stable template–monomer complex that can withstand the “harsh” conditions of polymerization and give good quality imprinted sites in the case of small organic templates. On the other hand, the interaction must not be too strong since using charged monomers to obtain a strong monomer–template interaction can lead to an undesirable effect, namely, a very high degree of non-specific binding. These monomers are randomly distributed over the surface of the polymer and in imprinting sites, resulting in a net negative or positive charge that can interact non-specifically with all species of the opposite charge. This problem can be solved by finding an optimal ratio between monomers and template molecules, which can be in silico determined using computer simulations [3,8,60,93,94]. Finally, in the case of protein imprinting, monomers with strong monomer–template interactions are not advisable because they can lead to many weak bonds between protein functional groups and neutral monomers, i.e., acrylamide, weak acids or bases [3,60].

## 3. Conductive Polymers for Electrosythesized MIPs

**Scheme 1:**
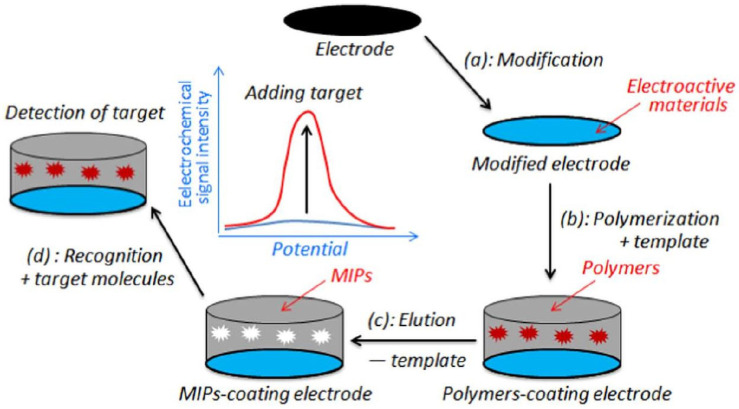
Workflow of the design and application of eMIPs-based electrochemical sensors. Reprinted with permission from [11]. Copyright 2018 Elsevier.

As mentioned above, bulk polymerization techniques have proven to be inadequate for imprinting macromolecules. Moreover, bulk MIPs for use in (bio)sensing applications are generally poorly compatible with the electrode, even if they have been previously ground [23]. This is due to a general problem of integration between binding sites or recognition units (i.e., receptor) and the transducer, together with the limitations of mass transfer and rebinding kinetics [23,95,96]. A plethora of creative strategies (mainly layer-by-layer (LBL)-based techniques) are continuously being developed and investigated to bring selective binding sites closer to the sensor surface and/or improve diffusion kinetics, ranging from spin-coating to other types of LBL assembly, grafting, and electropolymerization [23,97,98]. Electrochemical polymerization, or electropolymerization, seems to meet these requirements. Electropolymerization is a deposition process in which a polymer layer is formed or coated on a conductive substrate material in the presence of a template [59]. The electropolymerized MIP films shows excellent properties in terms of adhesion to the transducer surface, simplicity and fabrication speed [97,99]. Moreover, it allows in situ polymer synthesis from aqueous solution under mild conditions, which is ideal for protein imprinting, as well as easy control of morphology and film thickness [33,97,99]. The latter can be controlled by controlling the input current, resulting in greater reproducibility of the process than bulk polymerization [97,99,100]. The thickness of the polymer film correlates with the number of molecular cavities (i.e., binding or recognition sites), where thicker films have more imprinted cavities. However, these cavities may be too far from the surface, hindering access to the target [99,101]. The surface morphology can be tailored by selecting an appropriate solvent and supporting electrolytes. The swelling of the solvent and the interaction of ions in an electrolyte affect the stiffness and porosity of the electrosynthesized film [97]. The generation of eMIPs follows the same principles and steps as chemical (bulk) ones (Scheme 1). Briefly, a mixture of electroactive monomers and a template is polymerized directly on the transducer surface by controlling electrochemical parameters (current or potential) [59,97,99]. The electron flow converts many electroactive monomers into a conductive or non-conductive coating (depending on the type of monomer chosen) around the template. After subsequent removal of the template by electro-cleaning or with a suitable solvent, imprinted cavities remain in the polymeric network that are complementary in size, shape, and orientation of their binding sites to those of the template. In the method in which washing the MIP with a solvent is employed, the template must be sufficiently soluble in a solvent solution so that it simply detaches from the polymer network after a certain time with or without additional stirring. In the case of electro-cleaning, several potential sweeps on the MIP are performed using cyclic voltammetry (CV). The application of constant potential is also possible. In some cases, however, electro-cleaning may be unsuitable, because it can cause changes in the polymer structure. During incubation with an analyte, these cavities ensure selective binding of the target molecules with the eMIPs even in the presence of structurally induced interfering factors [97,99,102,103].

Although the electrosynthesis of MIPs is known to be a simple approach, the same challenges as with other polymerization techniques can arise:

- Template removal (i.e., “leaching” or “bleeding”), when it occurs, can damage the MIP (both conventional or electrosynthesized-based); this step often requires long term optimization;
- Unsatisfactory selectivity of the imprinted cavities, i.e., they may also interact with commonly present interfering analogues;
- No significant difference in the measured signal between eMIP and the corresponding non-imprinted polymer (i.e., a polymer prepared identically to eMIP without the addition of a template) [99].

It has been shown that an electrochemical approach is well suited for the preparation of protein-imprinted MIPs; most electroactive monomers can be deposited from the aqueous solution without affecting the native conformation of the protein. Moreover, the buffers that improve protein stability can be used as supporting electrolytes during the electropolymerization process. An advantage of electropolymerization over chemically initiated polymerization (with strong oxidizing agents) is that it can be carried out without an external initiator [32]. Depending on the choice of functional monomers and electropolymerization conditions (voltammetric, potentiostatic or galvanostatic polymerization), the as-fabricated polymer films can be conductive or non-conductive [32,59]. Electrically conducting polymers can be easily synthesized by electropolymerization in the presence of various counterions (i.e., doping ions), which allows easy modification of the properties of the resulting film. Their intrinsic conductivity results from the formation of charge carriers against oxidation (*p*-doping) or a reduction (*n*-doping) in their conjugated backbone [104]. Conducting polymer films can become thicker, whereas the insulating (nonconducting) film is self-limited. Namely, the insulating polymer film blocks electron transport between the transducer and the monomers once its thickness exceeds the range of electron transport (usually in the lower nanometer range), and/or the synthesized film is compact enough to prevent permeation of the monomer to the transducer [32,105]. Electrochemical techniques allow polymerization and doping to be performed simultaneously [106]. In addition, the sufficient biocompatibility of some conductive polymers [17] has stimulated interest in the development of suitable imprinted platforms for the detection of microorganisms and clinically relevant biomarkers, which are summarized in Table 2.

The most used electropolymerization technique for the preparation of protein-based eMIPs is CV. Using CV, the potential is periodically swept in the potential range where the monomer oxidizes and reduces, leading to polymer formation. During this process, the monomer on the electrode surface is subjected to cyclic (alternating between oxidative and reductive scans) regular changes in the applied potential within a potential window. This results in the formation of a conducting polymer film that alternates between the non-conducting (undoped) and conducting (doped) forms depending on the direction of the cyclic scan. By varying the scan rate and the number of scan cycles, the thickness and compactness of the deposited film can be controlled [32,59,118]. In general, slow scan rates form dense films with the template included. In contrast, too fast scan rates lead to loose polymer matrix formation characterized by low “molecular memory” [119,120].

The eMIPs can be developed via the potentiostatic route by applying a constant potential [59]. Initially, polymerization begins with the oxidation of the monomer on the electrode surface, which is characterized by an increased current density [65]. The growing thickness of polymer film can be controlled by the charge consumed during potentiostatic electropolymerization. The disadvantage is that this type of deposition does not control the resulting film’s compactness [32,121,122]. A polymeric matrix with refined adhesion to the electrode surface can be produced by applying a pulsed potential. In addition, the depleted monomer solution layer in the vicinity of the electrode can be replenished by the low diffusivity of the macromolecular template. This improves the incorporation of the protein template into the growing polymer film [32,65,107].

### 3.1. Polypyrrole (Ppy)

Due to its good biocompatibility and easy immobilization of various biologically active species, the polymer polypyrrole (Ppy) has one of the leading roles in electrochemical imprinting [17,23,32,97,123]. Preparation of a Ppy-based MIP system usually involves electrochemical oxidation. Removing oxygen from the monomer solution before polymerization may be required to achieve reproducible deposition of stable conductive films [123,124,125]. Application of more positive potentials (i.e., 1.0 V vs. Ag/AgCl(sat. KCl)) in buffer or the alkaline solution in the presence of oxygen can lead to further overoxidation of Ppy, which is often considered unfavorable [23,32,97]. This “overoxidation” is associated with polymer degradation and, consequently, loss of conductivity [32,97]. On the other hand, the partial degradation leads to the formation of additional functional groups, such as oxygenated carbonyl and carboxyl groups, which determine the semi-permeability of the synthesized polymer film and facilitate the selective recognition of a template [23,107,126]. Early attempts using Ppy-based electrochemical imprinting focused on the detection of anionic species due to the ability of the Ppy film to carry a positive charge [23]. It is expected that anionic templates can be extracted during the overoxidation of Ppy when the positive charge of the Ppy film is lost. Consequently, the imprinted cavities should remain in the overoxidized Ppy film, despite some structural changes in the imprinted film [127]. Since then, further development of the imprinting strategy has been explored using pyrrole (Py) as a functional monomer for the preparation of selective materials for amino acids (L-glutamic acid [128], L-aspartic acid [129], and L-tryptophan [130]), pharmaceutical compounds (paracetamol [131], sulfamethoxazole [132]), caffeine [133], ascorbic acid [134], and the mycoestrogen zearalenone [135]. The imprinting effect was achieved by forming hydrogen bonds and/or ionic interactions between some functionalities of the template and the Py units [23]. The possibility of modulating the number of imprinted cavities of the template via the MIP thickness with electrical stimuli has also been the subject of investigations [136]. The proposed mechanism, based on the electrochemical control of the degree of swelling of Ppy, is related to the ion transport of the polymer and provided foundations for the development of “smart” sensors.

The suitability of electrosynthesized Ppy for label-free detection of biologically active macromolecules, such as proteins [107,108] and hormones [137], was also demonstrated. For this purpose, a receptor for amperometric detection of bovine leukaemia glycoprotein gp51 present on the viral membrane was developed [107]. The signal of gp51 rebinding to the eMIP film was detected by pulsed (chrono)amperometry. Pulsed amperometry allows the detection of carbohydrates by measuring the current generated by their oxidation when voltage is applied [107,138]. Due to the carbohydrate component of the envelope glycoprotein gp51, the rebinding of gp51 was determined based on the calculated differences between anodic and cathodic peak currents in the obtained amperograms. The slow diffusion of the analyte to the electrode was reported, and the adsorption process was considered the rate-determining step. In this study, they were described by an exponential decrease to a minimum associated with the absorption of gp51 or, conversely, by an exponential increase to a maximum when gp51 was desorbed and/or extracted from the Ppy matrix by 1 M H_2_SO_4_ solution. Despite the promising results, this strategy did not consider the non-specific interactions and reusability of the Ppy film, which was mainly due to the 1 M H_2_SO_4_ used as a solvent for template extraction [107].

**Figure 4:**
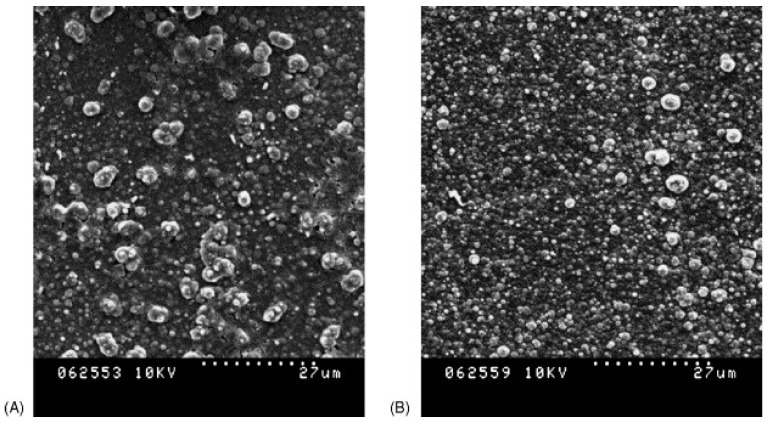
SEM micrographs of a PPy/poly(3-methyl thiophene: (**A**) non-imprinted film, (**B**) a *B. subtilis*-imprinted film. Reprinted with permission from [108]. Copyright 2007 Elsevier.

A study by Namvar and Warriner [108] provided a proof-of-concept for the design of microbially imprinted films using conductive Ppy and poly(3-methylthiophene) composite membranes for the determination of *Bacillus subtilis* endospores. Films (Figure 4) were prepared by adsorption of endospores onto the electrosynthesized Ppy, followed by deposition of a poly(3-methylthiophene) film with potentiostatic electropolymerization. The binding of *B. subtilis* endospores to the imprinted film was investigated by electrochemical impedance spectroscopy (EIS) by monitoring changes in susceptance. EIS is a non-destructive electrochemical technique that employs low-amplitude sinusoidal voltages in a certain frequency range. It offers several advantages over other electrochemical techniques, including determining relaxation processes. Accordingly, EIS can investigate intrinsic material properties or specific processes that could affect the conductivity, resistance, and capacitance of an electrochemical system [139,140]. EIS can be used to study the pore sizes of porous electrodes (by employing the appropriate equivalent electrochemical circuit) [140]. Due to these properties, EIS is a very important technique to study and understand the interfacial properties related to selective molecular recognition of bioactive molecules, or even whole cells. Magar et al. [139] claimed that the differences in the EIS response of the conductive polymer after the binding of the spores in the cavities (affinity sites) of the polymer were probably due to the realignment of the chains of the polymer network. These changes were also related to the proposed mechanism by which the binding of an antigen on the surface of antibody-immobilised Ppy alters the film’s conductivity. Spore absorption was also indirectly detected by following the germination of the bound endospores with CV. The underlying principle of this approach was to activate the germination of spores absorbed onto the surface conducting polymer films and measure the subsequent release of Ca^2+^ or dipicolinic acid (DPA) from the spore core. Due to the ionic nature of the mobility of Ca^2+^ and DPA, the spore absorption was determined by measuring the charge on the films following redox cycling. Namely, it was assumed that both Ca^2+^ and DPA could be involved in the ion-exchange transition of the supporting Ppy film between oxidized and reduced states. In this scenario, detection of absorbed spores was more sensitive in the presence of a chelating agent (ethylene glycol tetraacetic acid, EGTA) because the signal was enhanced by the interaction of the conducting polymer with Ca^2+^ or DPA released from the spore core during endospore germination. Although this work provided a proof-of-concept for the preparation of microbial imprinted films with conducting polymers, the regeneration of the MIP films without loss of their conductive activity was not possible [108].

Recently, electrochemical overoxidation of PPy was performed for the controlled release of cortisol in saliva samples [137]. The stepwise fabrication of the imprinted sensor was characterized by CV and scanning electron microscopy (SEM). The authors suggest that a sensor fabricated in this manner can be used for the in-field measurements of macromolecules, such as sterol hormones.

These efforts introduced more flexible non-covalent tactics to design innovative MIPs as artificial receptors with selectivity to biological macromolecules and microorganisms. However, there is still room for improvement in terms of binding and selectivity [23,97].

### 3.2. Polyaminophenyl Boronic Acid (PAPBA)

As a sensing material, polyaminophenyl boronic acid (PAPBA) can reversibly mediate the recognition of various macromolecules. It is known that boronic acid forms covalent bonds with diol-containing molecules (e.g., 1,2- or 1,3-diols), such as carbohydrates and proteins, at physiological or moderately alkaline pH [97,109,141,142]. In acidic solutions, the formed boronate esters dissociate. As an electroactive functional monomer, aminophenyl boronic acid (APBA) can be deposited on a transducer surface by oxidative electropolymerization. The boronic acid groups functionally attached to the polymer backbone can couple with cis-diol compounds. Therefore, APBA is a good candidate for the preparation of MIP-based films with homogeneous imprinted sites to detect cis-diol compounds [109]. Electrochemically prepared PAPBA-based MIPs have been used as sensing platforms for the detection of saccharides [28], proteins [142], and neurotransmitters [109].

A new electrochemical synthesis route of saccharide-templated MIPs from APBA was developed to detect fructose [28]. The method was based on the formation of a (D-fructose)-APBA complex within polyaniline (PANI) in the presence of fluoride (F_2_) by electrochemical deposition under slightly acidic (pH 5) or neutral conditions, which was deposited on the glassy carbon electrode (GCE). An intriguing feature of the self-doped polyaniline boronic acid, is that it can maintain high conductivity at elevated pH, allowing complexation between saccharides and aromatic boronic acids. The dual role of F_2_ was to (1) balance the conflicting pH requirements of aniline monomer (ANI) polymerization (usually at low pH) and boronic acid-saccharide complexation (usually at neutral or higher pH), and (2) to disrupt any B-N interactions between the 3-aminophenyl boronic acid (3-APBA) monomers. In the resulting self-doped MIP film, an anionic boronic acid ester complex was formed between 3-APBA and D-fructose. The D-fructose was removed from the polymeric network by soaking the MIP-coated GCE overnight in phosphate-buffered saline (PBS) at neutral conditions (pH 7.4). Since only self-doped polyaniline boronic acid is sufficiently conductive to allow continued polymerization at the polymer/solution interface, the authors investigated the electrochemical behavior of MIP self-doped polyaniline boronic acid by CV at lower pH in the presence of one molar equivalent of F_2_. They postulated that the F_2_ would enhance the electropolymerization of the saccharide complex with 3-APBA, simultaneously with the formation of the self-doped polymer. The obtained voltammograms showed that the addition of one equivalent of fluoride with respect to 3-APBA led to an efficient and sustained electropolymerization. This behavior suggested that both fluoride and D-fructose are needed in the polymerization, as neither F_2_ nor D-fructose alone at these concentrations are sufficient for electropolymerization. The ability to rebind D-fructose was evaluated by potentiometric measurements. The potentiometric response of the MIP film was selective for the D-fructose analyte even in the presence of D-glucose as a possible interferent. The authors found that the imprinted electrodes showed about a 25% increment in response compared to the non-imprinted electrodes, suggesting that imprinting in this manner can significantly affect the selectivity of complexation reactions with boronic acids [28,97].

The ability of electrodeposited imprinted PAPBA to reversibly mediate protein recognition has also been investigated [142]. The protein-imprinted electrode was constructed as a three-layer assembly of different conducting polymers by the voltammetric deposition on a screen-printed platinum electrode in the presence of target proteins (lysozyme and cytochrome *c*—cyt *c*). An initial layer of PPy was used as the supporting polymer layer, on top of which two additional PAPBA layers were added. A thin intermediate layer of PAPBA was non-imprinted and served as a barrier between the PPy and the outer protein-imprinted PAPBA layer. The function of PPy was to increase the absolute magnitude and sensitivity of the PAPBA films by overcoming the conductivity limitation introduced by the intermediate NH group linking two APBA residues. After template removal in acidic media (i.e., 3 wt.% acetic acid), the rebinding of the target protein was recorded using CV. The obtained anodic peak current showed a distinct two-phase binding profile for the rebinding of lysozyme and cyt *c*. In contrast, binding to the non-imprinted counterpart (without adding the protein template) showed progressive binding, characteristic of nonselective recognition. The decline of an anodic peak current of the polymer redox moiety was attributed to the binding of the non-conducting target protein to the polymer surface. This decrease in current was used to measure the extent of protein binding [97,142].

**Figure 5:**
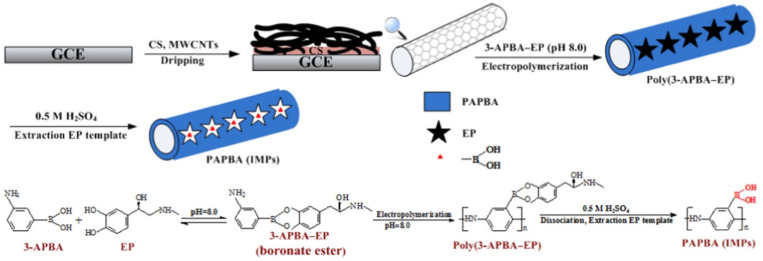
Graphical depiction of the constructed EP-imprinted biosensor preparation. Reprinted with permission [109]. Copyright 2019 Elsevier.

Recently, a nanocomposite with multi-walled carbon nanotubes (MWCNTs) coated with surface imprinted PABPA was developed for the sensitive detection of a catecholamine neurotransmitter, epinephrine (EP) [109]. The proposed MIPs biosensor (Figure 5) was constructed by electropolymerization of 3-APBA monomers on the surface of MWCNTs attached to a chitosan-coated GCE (MWCNT/CS/GCE).

The EP-imprinted cavities on the PAPBA matrix may be more selective for EP recognition since this catecholamine is inherently chiral. The positively charged character of the chitosan layer was exploited for homogeneous and stable assembly of the negatively charged MCWNTs using an LBL approach through electrostatic interactions between MWCNTs and chitosan. The EP-imprinted MIP film was prepared by potentiodynamic electropolymerization at slightly alkaline conditions (pH 8). The entrapped EP molecules were removed by both chemical and electrochemical routes. The template was first extracted in acidic media (0.5 M H_2_SO_4_ aqueous solution), followed by electrocleaning using CV. The morphology and properties of the sensing platform were characterized using SEM and EIS. The imprinted PAPBA layer, PAPBA(MIPs), contributed to a lower charge transfer resistance and improved electrochemical performance for epinephrine detection. The enhanced electrochemical response can be attributed to many imprinted cavities with the boric acid group, which can selectively adsorb epinephrine molecules and the synergistic effect between the MWCNTs and the PAPBA(MIPs) layer. Accordingly, under optimal conditions, the PAPBA(MIPs)-based electrode could effectively detect EP in the presence of many possible interfering factors (ascorbic acid, uric acid, and phenylepinephrine), applied as the coexisting compounds or structural analogues, due to the uniform distribution of the imprinted sites, identical in shape and size to the target. The unhindered detection of EP was also observed in the presence of monosaccharides, including glucose, fructose and mannose, with their cis-diol structure, which may interfere with EP detection. Moreover, the electrochemical detection of EP in human serum and real samples provided satisfactory results, making the imprinted PAPBA/MWCNTs nanocomposite a good candidate sensing platform for detecting catecholamines [109].

### 3.3. Polyaniline (PANI) and Related Compounds

Functional monomers of ANI [17,104,143,144,145] and a related compound, *o*-phenylenediamine (*o*-PD) [71,104,110,111,146], were also used for imprinting of various molecules (glucose, insulin, bovine serum, ascorbic acid, etc.). These were shown to be suitable for MIP preparation using ANI, since they possess functional groups that can participate in hydrogen bonding, π-π stacking, and other types of interactions with the template [17,32,110].

#### 3.3.1. Polyaniline (PANI)

Besides PPy, PANI is one of the most widely used polyaromatics due to its excellent and controllable chemical and electrochemical properties, including ease of processing, mechanical stability, intrinsic conductivity, hydrogen bonding, and redox sensitivity. These properties also contributed to PANI being the first commercially available conducting polymer [17,104,144,147]. However, some shortcomings of PANI, including insolubility, high brittleness, acid-catalyzed oxidative degradation, and especially electroactivity only in acidic media (pH < 4), limit its application in electrochemical sensing [104].

The electropolymerization of PANI can be carried out either at a constant current, constant potential or by potential sweeping [106]. The oxidative polymerization of ANI occurs in a wide pH range. At the same time, the mechanism depends on the acidity of the used medium, which also manifests in different properties of the resulting PANI. Polymerization at pH between 5 and 14 leads to the formation of polymer films with low conductivity. On the other hand, PANI films synthesized at highly acidic conditions (pH < 2) are characterized by high conductivity. In addition, the pH of polymerization also determines the magnetic properties of PANI and its solubility in organic solvents. PANIs electropolymerized in neutral and slightly acidic media are particularly interesting in protein imprinting because of their complex supramolecular structure [148].

Some attempts have been made for a generation of different PANI-based MIP sensors using different water-soluble templates, such as ascorbic acid [144] and bovine serum albumin (BSA) [145]. Roy et al. [144] used molecular imprinting technology to create imprinted PANI films deposited on an indium-tin-oxide (ITO) electrode to detect ascorbic acid. The molecular imprinted PANI electrode (AA-MI-PANI/ITO) was prepared by overoxidation of the ascorbic acid-doped PANI electrode, resulting in the removal of ascorbic acid from the PANI film. Characterization by Fourier transform infrared spectroscopy (FTIR), SEM, CV and differential pulse voltammetry (DPV) indicated the presence of ascorbic acid in the PANI matrix, which also served as a doping agent for PANI [144].

For highly sensitive and selective BSA determination in human serum samples, an imprinted PANI membrane was prepared and electrodeposited on a carbon electrode surface modified with a CNTs/graphene nanocomposite (CNTs/GP/CE). The superior electroanalytical performance over BSA can be attributed to both the CNTs/graphene nanocomposites with high electrochemical signals and the MIPs film with numerous selective recognition sites together with the fast diffusion of the hexacyanoferrate redox probe [145].

#### 3.3.2. Poly(o-phenylenediamine) (PPD)

The *o*-phenylenediamine (*o*-PD) is another electroactive monomer accepted in electrosynthesized imprinted polymers. The poly(*o*-phenylenediamine) (PPD) films grow compact and rigid, resulting in good mechanical stability and integrity with the possibility of hydrophilic or hydrophobic recognition sites. The electrochemical oxidation of *o*- PD, which is an irreversible process, can be characterized at various pHs (tested for pHs 1, 4 to 10, and 13 [149,150]) by a typical behavior of polymer films on the electrode surface blocking the access of the monomer to the electrode surface. This behavior indirectly indicates the formation of a very compact and insulating film that is essentially free of holes [151]. Since electrochemical polymerization allows control over the thickness of the polymer films, these films can be prepared thinly and continuously. This is of particular interest in developing enzyme-based biosensors [23,97,151,152]. It is known that an increase in the amount of entrapped enzyme can only be achieved by increasing the film thickness. However, this increases the reaction time, resulting in lower electrode sensitivity. In contrast, the PPD film with low thickness was able to trap a significant amount of active enzyme molecules, resulting in shorter response times [151]. Usually, they are electropolymerized via CV in aqueous buffered solutions at various pH values, mainly in acetate buffer at pH 5.2. Similar to PANI, the polymerization pH affects the conductivity of PPD films; at slightly acidic conditions (pH 5.2), the PPD film exhibits a non-conductive character, which is useful for designing MIP sensors based on capacitance changes [23]. Several types of PPD-based biomimetic sensors have presented the possibility of using *o*-PD as a functional monomer for clinically relevant molecules such as glucose [152], insulin [71], butyrylcholinesterase (BuChE) [110], and vascular endothelial growth factor (VEGF) [111].

This polymer was the first case of an electrosynthesized MIP film for a neutral template, glucose [152]. The commonly used Py is unsuitable for imprinting neutral molecules, such as glucose, because the resulting imprinted PPy film must be overoxidized to lose the positive charge [127,152]. Due to the above-mentioned properties of PPD films, the *o*-PD monomer was chosen instead for glucose imprinting by potentiodynamic electropolymerization under improved selectivity and sensitivity conditions. After electropolymerization, the template (glucose molecule) was removed through a washing procedure with triply distilled water. The affinity of the imprinted PPD film for glucose and the number of binding sites were determined by Scatchard analysis of the calibration curve, a tactic already used in MIP-based work [152,153]. It was estimated that binding sites involved about two *o*-PD monomers for each glucose molecule. This low ratio could be partly due to the coexistence of some specific adsorption/permeation in the deposited PPD film. The obtained Scatchard plot revealed two types of molecular cavities for the low and high glucose concentrations. Glucose was strongly bound in the imprinted cavities at lower concentrations, while at higher concentrations, it was also located within the polymeric network where it was weakly bound [97,152].

As insulin plays a key role in controlling glucose homeostasis, it is an important biomarker for diabetes. Therefore, measuring insulin levels in diabetic patients is important for a better prognosis [154]. For this purpose, a molecularly imprinted PPD film containing insulin was prepared based on epitope imprinting [71]. In this work, the C-terminal polypeptide of insulin (C-insulin polypeptide) was used as a template molecule that self-assembled directly on the surface of an Au electrode, followed by electrochemical polymerization of an insulating layer of *o*-PD by CV. The template was removed by immersion in a NaOH solution, leaving molecular cavities that selectively detect insulin. This imprinting insulin sensor enabled the re-adsorption of insulin, which was indirectly detected by DPV using hexacyanoferrate as a redox probe. The C-insulin polypeptide as a template reduced the steric hindrance of the insulin recognition process and simplified template removal.

**Figure 6:**
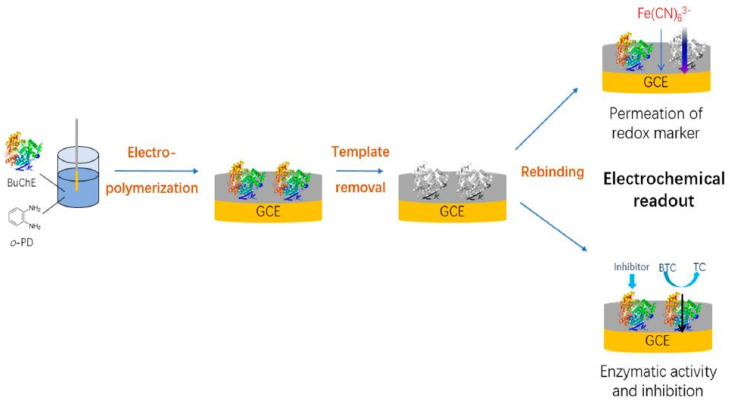
Schematic representation of the preparation and electrochemical characterization of the BuChE-MIP sensor. The MIP layer was formed by electropolymerization of *o*-PD in the presence of BuChE on the surface of glassy carbon electrodes, followed by incubation in 100 mM NaOH to remove the template. All steps of MIP synthesis and re-binding of BuChE were evaluated by measurement of ferricyanide by CV and amperometric measurement at 0.4 V of thiocholine formed during BuChE-catalyzed conversion of butyrylthiocholine iodide (BTC).

The wide range of templates, from low molecular weight (drugs and pesticides) to high molecular weight (proteins) in *o*-PD-based MIPs, indicates that hydrogen-bonding, π-π and other types of interactions are sufficient for binding very different substances. Ozcelikay et al. [103] recently designed the first MIP to detect the diagnostically relevant enzyme BuChE (Figure 6).

The proposed BuChE-based MIP was electrochemically synthesized by the oxidative potentiodynamic polymerization of *o*-PD and deposited as a thin film on a GCE. All steps of the MIP process, the electrosynthesis of MIP (electropolymerization, removal of the protein template from the polymer in alkaline solution), and the rebinding of BuChE were characterized by CV using hexacyanoferrate as a redox probe. The enzymatic activity of BuCHe was measured by amperometry. Both methods allowed measurements of BuChE in the picomolar (pM) range on the MIP-based sensor, as prepared without signal amplification, and achieved the sensitivity of immunoassays. For measurements in this sub-nanomolar concentration range, the signal is generally amplified by adding NPs, nanotubes, graphene, or repeated binding and dissociation. In this study, the electrochemical readout was achieved without using the above strategies. However, cross-reactivity of the MIP sensor with abundant components in the blood (e.g., serum albumin) affected the measurement of BuChE in blood samples since serum albumins have an almost 10,000-fold excess in relation to the enzyme BuChE. Nevertheless, the authors showed that measurement of the enzymatic activity of the biocatalyst allowed direct quantification of rebinding at the MIP sensor surface. This was demonstrated by measuring the anodic oxidation of thiocholine, the reaction product of the enzymatic conversion of butyrylthiocholine iodide (BTC). The increase in current after the addition of BTC reflected the activity of BuChE bound to the MIP. The latter resulted in a low LOD of 14.7 pM. In addition to low LOD due to local substrate formation, the assay combined synergistic recognition by the MIP and substrate selectivity of the target enzyme (BuChE), due to the spatial arrangement of the interacting groups and the shape of the cavities.

Cancer is one of the deadliest diseases of the present day. Early diagnosis of cancer is critical to the chances of recovery, as most forms of cancer cannot be diagnosed until metastasis has already occurred. There are many different biomarkers associated with different types of cancer that can be used for the early diagnosis of the disease, understanding pretreatment conditions, and tracking treatment efficacy [4,111]. Among them, VEGF, which is responsible for angiogenesis in wound healing, diabetic retinopathy, and rheumatoid arthritis, can be used as a biomarker for solid tumor growth in various cancers. A sensitive label-free sensing platform has been developed for VEGF [111] based on MIP as a biomimetic receptor combined with graphene screen-printed electrodes (GSPEs). The VEGF-based MIP sensing assembly was constructed by potentiodynamic polymerization of an *o*-PD monomer around a VEGF molecule on GSPEs using CV, followed by solvent extraction of a template with 0.25 M NaOH solution in ethanol: water (2:1 *v*/*v*). They used EIS as the methodology for the analysis. This impedimetric sensor had good sensitivity and reproducibility for label-free VEGF detection, with a linear response in the range from 20 to 200 pg/mL and a LOD of 0.08 pg/mL, which met clinical requirements. Furthermore, the electroanalytical performance of the sensor was also evaluated in human serum samples spiked with VEGF, and the results obtained indicate its potential application in real sample measurements.

**Figure 7:**
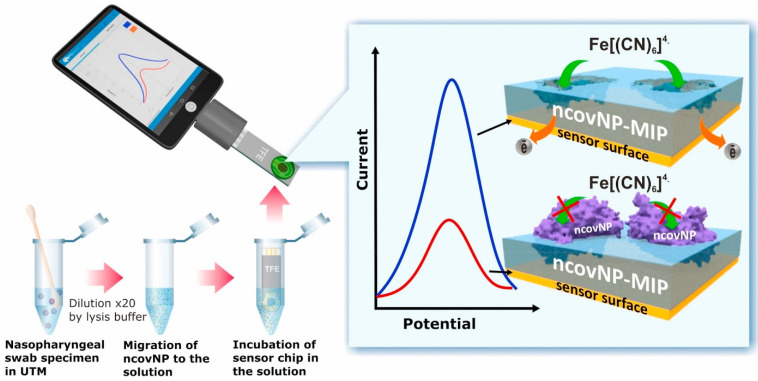
Concept of the clinical feasibility of the nconNP sensor analyzing the samples prepared from nasopharyngeal swab specimens. Reprinted with permission from [112]. Copyright 2021 Elsevier.

In the context of the current COVID-19 pandemic, Raziq et al. [112] presented a rapid COVID-19 portable electrochemical sensor (Figure 7) based on MIP that is selective for SARS-CoV-2 nucleoprotein (ncovNP).

A key element of the sensor is a disposable sensor chip in the form of a thin film electrode (TFE) equipped with MIP-enriched selectivity for ncovNP. The ncovNP-imprinted MIP film was prepared from poly-*m*-phenylenediamine (P*m*PD), deposited on a gold TFE (Au-TFE) surface. The use of *m*-phenylenediamine (*m*PD) as a suitable functional monomer was determined using computational modelling. The protocol for the synthesis of ncovNP-MIP was adapted by Tretjakov et al. [155]. Briefly, the ncovNP-modified sensing surface was exposed to the synthesis solution. The electrosynthesis of PmPD on ncovNP-modified Au-TFE, which served as the working electrode, was performed by applying a constant potential of 0.6 V to the working electrode. Molecular cavities of ncovNP were generated by treating the polymer film with an ethanolic solution of 2-mercaptoethanol to release covalently bound ncovNP. Each step of the preparation procedure was characterized by CV, whereas the rebinding of the target analyte was studied by DPV in the presence of a redox couple. The prepared ncovNP-imprinted sensor showed a linear response to ncovNP in the lysis buffer up to 111 fM with a LOD of 15 fM. Moreover, the clinical feasibility of the developed ncovNP sensor was tested by analyzing nasopharyngeal swab samples from patients. Satisfactory results indicate a viable route for constructing rapid COVID-19 diagnostic tools using this technology [112].

### 3.4. Polyphenol-Related Compounds

Phenol and its derivates are widely used as recognition units for MIP-based sensors because they are easy to prepare and can interact with many different analytes through π-π interactions [97]. The electrooxidation of phenol occurs by forming a phenoxy radical, which can react with other species present in the solution to form products or react with other phenol molecules to form a dimeric radical [116]. One of the first successful electrosynthesized MIPs was based on imprinting polyphenol for phenylaniline detection, which led to the development of the first capacitive sensor with a synthetic artificial receptor layer MIP. In particular, phenols bearing an amino group are considered efficient materials for electrosynthesized MIPs due to their ability to form functionalized molecular cavities. As such, they can provide a more selective interaction with a template molecule [23]. An *o*-aminophenol-based MIP is favored as an artificial recognition element because it offers several advantages over other electroactive polymers. Like others, *o*-aminophenol can be electropolymerized in situ on various transducers, and the polymer thickness can be controlled within 10–100 nm due to its self-limiting growth. In addition, the poly(*o*-aminophenol) film can be easily regenerated after use by changing the applied potential to a conducting polymer film. In this way, the counterions can be either inserted or ejected to maintain electroneutrality. The movement of the counterions can also cause the transfer of neutral species, such as solvent molecules, into or out of the film [114,156]. Another unique feature of the poly(*o*-aminophenol) film is the electron-donating hydroxyl group, which has two functions: (1) positioned next to the imine nitrogen, it increases the electron density at the imine sites; (2) it is itself a potential coordination site [114]. Phenol-derived functional monomers have been used to design molecularly imprinted polymers for a diverse range of pharmaceutical and clinically relevant compounds, such as antibiotics [23,100], 3-nitrotyrosine (3-NT) [116] neurotransmitters, such as dopamine [113] and norepinephrine (NE) [114], and cancer biomarkers [90,113,114].

Increasing research interest in the electrochemical imprinting of *o*-aminophenol led to the construction of sensing devices for dopamine [113] and NE [114]. A first attempt of using *o*-aminophenol in constructing an MIP sensor was demonstrated for dopamine [113]. Dopamine is a natural neurotransmitter that plays an essential role in controlling the central nervous system, cardiovascular, renal, and hormonal functions. It is also associated with drug addiction and Parkinson’s disease. As a neurotransmitter, dopamine has electrochemical activity. However, in biological tissues, its electrochemical detection is essentially prevented by high levels of ascorbic acid. Using molecular imprinting principles, Li et al. [113] strived to fabricate a highly selective and sensitive electrochemical sensor for dopamine detection. The MIP detection layer was constructed by potentiodynamic electropolymerization of *o*-aminophenol monomers around the dopamine molecule on the surface of an Au electrode. Dopamine was chosen as the template molecule because of its prevalence and electroactivity mentioned previously. After electropolymerization, the template was chemically removed in an acidic medium (0.5 M H_2_SO_4_). Under strongly acidic conditions, dopamine molecules can be released from the molecular cavities due to the destruction of hydrogen bonds between the template and the *o*-aminophenol monomers, resulting in an electrode with a dopamine-imprinted layer. The electrochemical response of the proposed MIP sensor to dopamine was evaluated using CV and DPV to check the changes in oxidative currents of a hexacyanoferrate, which served as a redox probe. The obtained results showed that the imprinted poly(*o*-aminophenol) recognition unit could selectively rebind its respective template molecule, even in the presence of a high concentration of the ascorbic acid as an interferent, which prevents an electrochemical detection of dopamine in biological samples due to a large overpotential for dopamine oxidation with conventional electrodes [113,157,158]. Overall, under optimized conditions, the linear concentration range of dopamine from 20 nM to 0.25 µML with an LOD of 1.98 nM [113]. The electrosynthesis of *o*-aminophenol was further used to construct a voltammetric sensor for NE detection [114]. NE, a catecholamine neurotransmitter, plays an important physiological role in the function of various organ systems (renal, endocrine, cardiovascular, central nervous, and reproductive). Among other stimulants, NE is also on the World Anti-Doping Agency’s 2005 list of prohibited chemicals, so many attempts have been made to determine NE quickly and reliably. In this regard, the *o*-aminophenol monomer with NE was electropolymerized on the surface of a GCE by CV to prepare a catecholamine-imprinted polymer. The similarities in the methodology of MIP generation with the aforementioned one also includes the conditions used to remove the template (dipping an imprinted sensor in 0.5 M H_2_SO_4_ overnight). Strongly acidic conditions break the hydrogen bonds between the template molecules and the functional monomers, resulting in the NE-imprinted polymer layer. The fabricated sensor was used to monitor NE content in pharmaceutical and biological samples, such as human blood and urine, using square-wave voltammetry (SWV) and a standard addition method. SWV is widely used to decrease the LOD. Compared with other pulsed voltammetric techniques, such as DPV or normal pulse voltammetry, SWV allows shorter analysis times [159]. Coupled with the SWV method, the imprinted sensor displayed a linear relationship between current response and NE concentration. The authors developed a sensor with an improved LOD (0.49 nM) compared with other similar studies published in recent years. In addition, the proposed MIP-based sensor showed a high degree of selectivity for the target molecule compared with other interfering factors, including ascorbic acid and uric acid, present in biological fluids. It was also characterized by long-term stability, good reproducibility of recognition sites for norepinephrine, and high regeneration ability of the imprinted cavities [114].

Breast cancer is one of the most commonly diagnosed cancers, and its incidence has increased over the years [4,90,115]. Despite achievements in screening programs and treatments, it is still the leading cause of cancer-related mortality in women. Significant studies have been conducted to identify appropriate biomarkers for breast cancer. Among them, the cancer antigen 15-3 (CA15-3) has been routinely adopted as a potential predictor of treatment failure in the metastatic stage. As a good candidate for use in point-of-care (POC) devices, Pacheco et al. [90] developed a sensing biomimetic platform for the breast cancer biomarker CA15-3. They modified an Au screen-printed electrode (AuSPE) with an MIP layer. They used 2-aminophenol as the functional monomer, forming a non-conductive polymeric network. In conjunction with electroanalytical detection methods, non-conductive polymers usually narrow the working linear range of a system [4,90]. The imprinting procedure involved two steps: (1) adsorption of CA15-3 onto AuSPE; and (2) electropolymerization of 2-aminophenol around the adsorbed protein. After template removal by acid solvent extraction (using 0.5 M oxalic acid), the sensor was characterized by voltammetric techniques, including CV, DPV, and EIS, using the hexacyanoferrate redox probe. The authors found significant differences when comparing CVs of electropolymerization between the non-molecular-imprinted (without template molecule) polymer (NIP), as a control, and MIP. In the case of NIP, the oxidation peak of 2-aminophenol, indicating the formation of a non-conducting film, appeared only in the first cycle. In the case of MIP, the oxidation peak of 2-aminophenol was also observed during the first cycle, but it decreased more slowly in the subsequent cycles. This electrochemical behavior during the MIP electropolymerization process is probably due to the adsorbed protein, CA15-3, on the surface of AuSPE, which slows down the formation of the nonconducting film. The as-prepared sensor showed a linear correlation between the peak current height of the hexacyanoferrate and the logarithm of CA15-3 concentration in an operating range of 5-50 U/mL. Moreover, the LOD reported (1.5 U/mL) was lower than the cut-off value in clinical practice (25 U/mL), making the developed MIP-based sensor a promising tool for clinical purposes [90].

Pacheco et al. [115] reported a similar system as the one mentioned above for detecting another breast cancer marker, an extracellular domain of human epidermal growth factor receptor 2 (HER2-ECD), using similar strategies to design an MIP-based sensor and electroanalysis. In this work, they used phenol as an artificial recognition moiety, which was electrosynthesized by CV using a pre-polymerization solution containing phenol and HER2-ECD, resulting in the formation of a polyphenol film with entrapped HER2-ECD molecules. During electropolymerization, the typically irreversible redox process of phenol was observed, and as the number of scans increased, the oxidation current intensity decreased. The latter indicated an increased formation of a polymer film on the surface of AuSPE, since polyphenol is a non-conducting polymer that hinders electron transport to the electrode surface. After template removal by solvent extraction, the imprinted voltammetric sensor was characterized by CV and EIS, while electrochemical detection of HER2-ECD was performed by DPV using hexacyanoferrate as a redox probe. In the analysis of other protein biomarkers (CA 15-3, renal protein cystatin C), the MIP sensor showed selectivity for the HER2-ECD biomarker. Moreover, the obtained results from sampling HER2-ECD in human serum indicate that the developed MIP sensor is a promising tool for early clinical diagnosis and follow-up in addition to the possibility of rapid detection and decentralized analysis.

**Figure 8:**
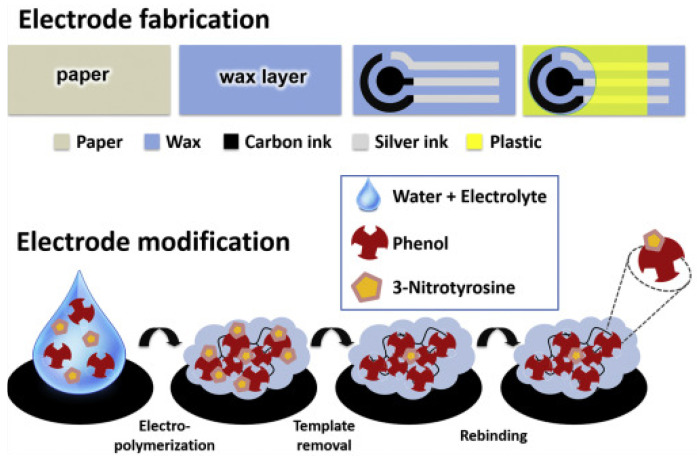
Schematic presentation of designing a paper-sustained device for label-free detection of 3-NT. Reprinted with permission from [116]. Copyright 2020 Elsevier.

Recently, phenol has been used to tailor an MIP material for the label-free detection of 3-NT [116], which is postulated to be a relevant biomarker for oxidative stress (OS). At OS, reactive oxygen species (ROS) are overproduced, causing cellular damage in proteins, deoxyribonucleic acid (DNA), and lipids. As for the latter, 3-NT is a subproduct formed when proteins are attacked by free radicals, and it has been linked to chronic disease induction as a biomarker of OS. A novel 3-NT-imprinted MIP electrochemical sensor (Figure 8) is the first representative of a paper-sustainable device. The biomimetic material was directly assembled on a paper platform, made conductive with carbon ink and, as such, rendered suitable for electrochemical transduction. Electrosynthesis of the polyphenol film in the presence of a template (3-NT molecule) was carried out by potentiodynamic electropolymerization, followed by removal of the template in methanol/water solution. The electrochemical behaviour of the constructed MIP sensor was characterized by CV and EIS using a hexacyanoferrate redox probe. The proposed sensing system utilized DPV as the electroanalytical technique. The sensing system showed good electroanalytical performance in the oxidation of 3-NT biomarkers in human urine samples in terms of sensitivity, selectivity, and reproducibility. The inclusion of a tailored in situ MIP enabled the efficient determination of a targeted biomarker in complex samples. The presence of species oxidizing near the oxidation potential of 3-NT contributed to the final response.

### 3.5. Polyscopoletin

Scopoletin, a coumarin derivate, offers some improved properties compared with PPy, such as good monomer solubility in aqueous solutions, the hydrophilicity of the deposited polymer, and the simplicity of its electropolymerization. The latter is robust and does not require deoxygenation of the monomer solution [32,160]. Furthermore, it can be electropolymerized at low oxidation potential (0.4–0.7 V vs. Ag/AgCl), and under such circumstances, the electrochemical removal of a protein template from an electrode surface is negligible. Scopoletin provides functional groups that can participate in hydrogen bonds, van der Waals forces, and hydrophobic interactions with the template [161]. Therefore, scopoletin as a functional monomer has proven to be very efficient for MIP electrosynthesis, especially for protein imprinting [162]. Although satisfactory results have been obtained with polyscopoletin-based MIPs using a variety of protein templates, such as cyt *c* [117], heme protein [163], and tyrosine [146], this polymer remains an underestimated material in the field of imprinting [162].

Bosserdt et al. [116] constructed an MIP capable of binding cyt c with a defined orientation by combining epitope and electrochemical surface imprinting. The electrostatic attraction of the positively charged lysine residues in the adjacent heme group of cyt *c* can be adapted to bind to electrode surfaces through an anionic self-assembled monolayer (SAM), analogous to the reaction of proteins in the respiratory system. Oriented binding of cyt *c* to the electrode surface facilitates direct electron transport (DET) with the underlying electrode. To enable DET, a non-conductive polyscopoletin was electrodeposited from an aqueous solution containing scopoletin monomers and cyt *c* on the surface of an Au electrode, previously modified with mercaptoundecanoic acid (MUA). The MUA layer contributes to the electrostatic adsorption of cyt *c* by increasing the surface concentration of cyt *c* during the electrodeposition step and is crucial for the effective orientation required for DET. After electropolymerization using amperometry, the template was removed from the polymeric network under acidic conditions, leaving binding sites in the hydrophilic polymer film. The electroanalytical behavior of the as-prepared MIP electrode was investigated using CV. Measurement of the peak area and formal potential from the cyclic voltammogram allowed a simple estimation of the electroactive surface concentration and the nature of rebound cyt *c*. This is due to the presence of heme (iron porphyrin) in the cyt *c*, which changes its redox state (between Fe^2+^/Fe^3+^) during electron transfer in the respiratory chain. The same redox process occurs in cyt *c* molecules adsorbed on MUA-modified electrodes, with the heme group oriented towards the Au electrode. Studies of competitive binding with other proteins (BSA, myoglobin, lysozyme) showed that the polyscopoletin-based MIP preferentially binds its target molecule. The molecular shape and charge of the protein also determine the binding of interfering proteins. Moreover, the MIP-bound cyt *c* showed DET and pseudo-peroxidative activity.

This imprinting approach was further explored by Peng et al. [163]. They fabricated an MIP layer that encompassed DET and biocatalytic ability of the heme protein (hexameric tyrosine-coordinated heme protein, HTHP). Thin MIP films for the rebinding of HTHP were prepared by electrodeposition of polyscopoletin film after oriented assembly of HTHP on previously MUA-modified Au electrode. Cavities, complementary in shape and size to HTHP, were formed after template removal with acidic treatment. Rebinding HTHP to MIP was achieved by electrostatic attraction of the protein by a self-assembled monolayer (SAM) and molecular recognition by non-covalent interactions with the MIP. The quasi-reversible DET of HTHP rebinding was reflected by a pair of well-defined redox peaks in the cyclic voltammogram. The biocatalytic nature of the proposed MIP-electrode was demonstrated by its ability to catalyze the cathodic reduction of peroxide.

A polyscopoletin film also served as a polymeric network of a biomimetic sensor assembly for tyrosinase, one of the biomarkers for skin cancer [146]. A polyscopoletin film with entrapped tyrosinase molecules was deposited on the surface of an Au electrode by multistep amperometry. After electropolymerization, the template was removed enzymatically with proteinase K. Hexacyanoferrate was used as a redox probe to characterize the permeability of the MIP layer after each step of the process using CV. After electropolymerization, the current for the redox probe was almost completely suppressed due to the formation of the non-conductive polyscopoletin film. After template removal, the MIP-modified electrode showed a significantly increased hexacyanoferrate signal, indicating the formed molecular cavities through which hexacyanoferrate can diffuse. Subsequent incubation with tyrosinase resulted in a suppressed hexacyanoferrate signal because the cavities were filled with a non-conductive protein.

Recently, an imprinted polyscopoletin was used for the impedimetric detection of lysozyme [162]. Lysozyme is a potent antimicrobial enzyme that is abundant in nature. Due to its properties, it has found its place in food (in cheese ripening, brewing, and winemaking), pharmaceuticals (in the treatment of ulcers and infections), and in a clinical setting. Changes in lysozyme levels can indicate pathological conditions; for example, monitoring lysozyme levels allows differentiation between acute myelogenous or monocyte leukaemia and acute lymphatic leukaemia, and a tracking response to treatments in cancer patients. Two strategies were employed to electrodeposit a lysozyme-imprinted polyscopoletin film onto an Au electrode: (1) use of a monomer-template pre-polymerization mixture; and (2) covalent immobilization of the enzyme by a self-assembled anchor layer before polymer electrosynthesis. Each step was evaluated using CV and EIS. In agreement with the published work by Dechtrirat et al. [161], the MIP layer prepared by the second approach showed higher sensing performance in terms of selectivity, LOD, and reproducibility. Moreover, the measurements performed in artificial saliva provided satisfactory results, indicating a potential application of the MIP impedimetric sensor in a clinical setting.

## 4. Electrochemical Readout

**Figure 9:**
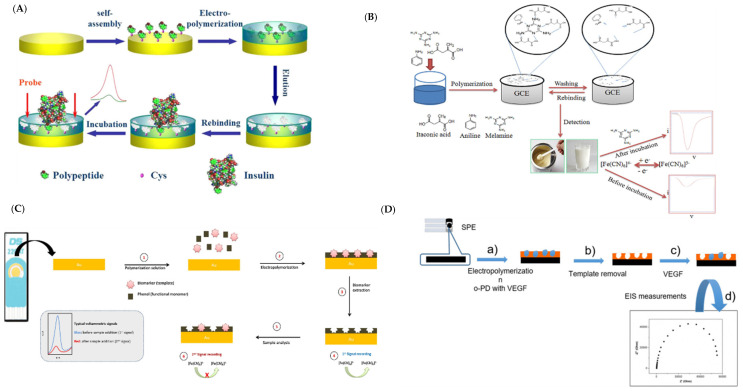
Electrochemical methods as a tool for preparation of MIP-layers and detection of target molecules: (**A**) preparation of an insulin MIP sensor based on epitope printing, reprinted from [71]; (**B**) the procedure of MIP nanocomposite-based sensor preparation and subsequent detection of melamine rebinding using SWV, reprinted from [106]; (**C**) construction of MIP electrochemical sensor for HER2-ECD and corresponding DPV measurements, reprinted from [115] (all three references with permission from Elsevier B.V.); (**D**) VEGF MIP preparation by electropolymerization and EIS-based recognition, reprinted with permission [111]. Copyright 2019 Wiley-VCH Verlag GmbH and Co. KG.

Electrochemical methods provide a sophisticated generation of the MIP layer directly onto the electrode surface and are potent tools for signal generation. The generation of the measuring signal from MIP-based sensors shares some aspects with immuno-sensing. Still, intrinsic effects in the polymeric network can interfere with the readout of the recognition in the molecular cavities (i.e., binding sites). Nevertheless, MIP electrochemical sensors are popular for directly quantifying redox-active analytes, determining redox enzymes or enzyme mimicking species by monitoring the formation of electroactive products in an “effortless”, productive, and highly sensitive detection strategy [2,7,9,32,33,97]. A commonly used detection approach is to monitor the permeability of a low-molecular-weight redox probe through thin MIP films. The simplified model for electrochemical signal generation assumes that the template removal creates pathways in the dense polymer matrix that allows the permeation of the redox probe at the electrode surface to provide the current signal through its oxidation or reduction. Re-bonding of the target subsequently suppresses the current signal by closing the imprinted cavities and, thus, the pathways to the electrode surface. In this way, a smaller amount of the redox probe molecules reach the electrode surface, resulting in a suppressed signal that is concentration-dependent [15,33]. This is a conventional electroanalytical mode for insulating MIPs that regulate the redox response of the probe species to the electrode surface. Since this permeability can be tracked by many electrochemical techniques, such as CV, SWV, DPV, and EIS (Figure 9), it can be adapted for the readout of MIP-based affinity sensors for proteins. It also enables a mode to characterize each step of MIP synthesis and evaluate the concentration dependence of target rebinding to the MIP.

**Figure 10:**
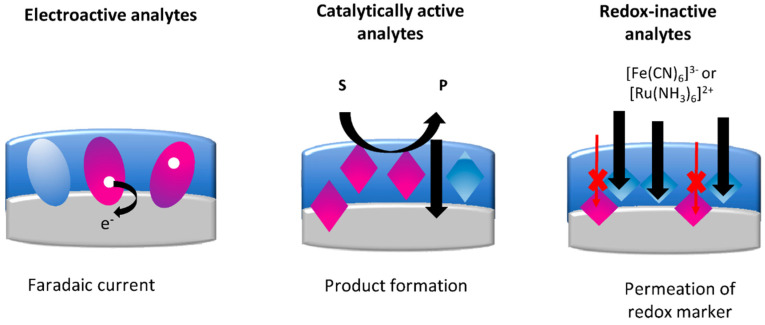
Three main approaches for achieving the electrochemical readouts of MIP-based sensors: a direct electron transfer (**left**), enzymatic activity (**center**), and the flux of a redox probe (**right**).

The electrochemical approaches for the electrochemical readout of MIP-based sensors can be divided into three categories (Figure 10) [7,33]:

1. The flow of a redox probe: the signal, regulated by the target binding, is detected at the underlying electrode surface;
2. Enzymatic activity: enzymatic activity is detected by the formation of a redox-active product at the underlying electrode; this pathway applies to catalytically active targets (enzyme targets, or enzyme-labelled targets, catalytically active MIPs);
3. Direct electron transfer (DET): faradaic current is recorded due to DET between the redox-active target and the underlying electrode.

### 4.1. Redox-Active Analytes

The most exact evidence of rebinding to the MIP is the electrochemical conversion of the target. The signal occurs when the target reaches the electrode surface and is based on DET between a target and the underlying electrode [7,33,117,163]. The lack of selectivity is not necessarily related to the insufficient selectivity of the molecular cavities. Still, it can also be attributed to nonselective voids in the polymeric network that formed in situ during preparation. On this basis, the MIP film is a molecular filter that differentiates the sample components according to the size and shape of the molecules. This separation leads to a significant increase in selectivity compared with the bare electrode. On the other hand, the partial blockage of the electrode surface by the formed polymer layer reduces the sensitivity compared with the unmodified electrode. This blockage can be overcome by integrating nanomaterials (NPs, CNTs, or graphene, particularly reduced graphene oxide) into the MIP, increasing the active surface area and thus enhancing the sensitivity [7,102].

The measuring signal can be actualized using bioelectrocatalysis, established on DET between the electrode surface and the redox protein target [33], such as haemoglobin [164]. This measuring concept is limited to external redox enzymes with surface-exposed redox centers that allow electron exchange with electrodes without the need for soluble mediators [7,165]. This principle was first demonstrated with the developed MIP for cyt *c* [117], and the same procedure was used to synthesize the MIP around a more complex protein, HTHP [163].

### 4.2. Catalytic Active Analytes

In the case of biocatalysts, including enzymes and enzyme-labelled analytes, template removal and rebinding can be quantified by estimating the biocatalytic activity of the MIP-based sensor. This has been successfully conceptualized for the electrochemical detection of various enzyme molecules (BuChE, tyrosinase). The surface activity sums the substrate conversion by the enzyme molecules within the imprinted cavities, together with the non-selectively adsorbed enzyme on the non-imprinted polymer surface [7,110,146]. Alternatively, the generation of the catalytic current upon addition of the (co)substrate requires that the protein target moves in a DET-“productive orientation” to the electrode surface [7,33,163].

The use of enzyme-labelled tracers can improve the electroanalytical performance of an MIP-based sensor. Such application in competing configurations offers the possibility of extension to electroinactive analytes. However, there are two considerations regarding such testing: (1) higher cost of measurement due to reagent costs, and (2) the enzyme may hinder the interaction of the target with the imprinted cavities, or it may interact with the polymer surface [7].

An extension of MIPs that mimic antibody function are MIPs that mimic enzymatic activity, developed by Wulf [7,49]. The theory behind the synthesis of catalytic MIPs is based on using the analogue transition state during the catalyzed reaction as a template [7,104]. Productive catalysis was obtained for the cleavage of esters, carbonates, and carbamates. The resulting MIPs can mimic catalysis by hydrolases. In contrast, incorporating metal ions or metal complexes into the polymer matrix of the MIPs enables oxidoreductase-like activity. The measuring signal is generated by displaying an electroactive product or the consumption of a (co)substrate such as oxygen or peroxide [7].

**Figure 11:**
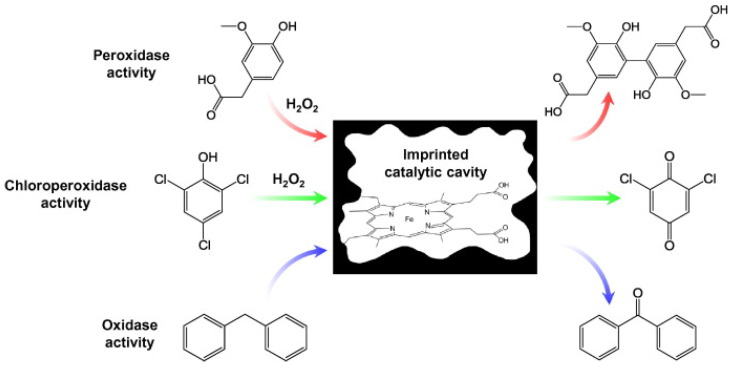
Catalytic activity of catalytic MIP with different substrates. with permission from [166]. Copyright 2012 Elsevier.

More straightforward than the use of metal complexes is the inclusion of redox-active enzymes into the polymer matrix to synthesize catalytic MIPs. For this purpose, hemin, the active site of peroxidases and cytochrome P450 enzymes, is often used (Figure 11) [7,166].

Coupling the catalytically active MIPs with the electrochemical sensing platform ensures a new line of creativity to synthesize advanced MIP-based sensors in terms of sensitivity, robustness, and ease of fabrication [7].

### 4.3. Redox-Inactive Analytes

Regardless of the electroactive nature of the target analyte, the most commonly used approach to characterize MIP sensors is to evaluate the diffusion permeability of a hexacyanoferrate redox probe using electrochemical techniques. However, the method of obtaining the electrochemical readout for redox-inactive species can vary. The attractiveness of this measurement method lies in its simplicity, cost-effectiveness, and high sensitivity. As mentioned before, it allows us to characterize each step of the MIP synthesis and to monitor the target rebinding to the MIP for low-molecular-weight targets, macromolecules and even (nano)particles [7,33].

In the case of low-molecular-weight targets, the imprinted cavities in the polymer matrix have a similar diameter to the redox probe. Therefore, several mechanisms have been proposed to affect the target’s rebinding to the current probe signal intensity, including changes in the porosity of the MIP layer or the diffusion rate of a probe, doping–“dedoping” effects, and changes in the electrical bilayer [7]. For macromolecules, the model of mechanisms predicts the formation of imprints after template removal in the tight MIP layer which, in turn, increases the permeation of the redox probe to the electrode surface. Rebinding of a target leads to shrinkage of the imprinted sites, which decreases the permeation of the probe [7,32].

## 5. Gate Effect

**Figure 12:**
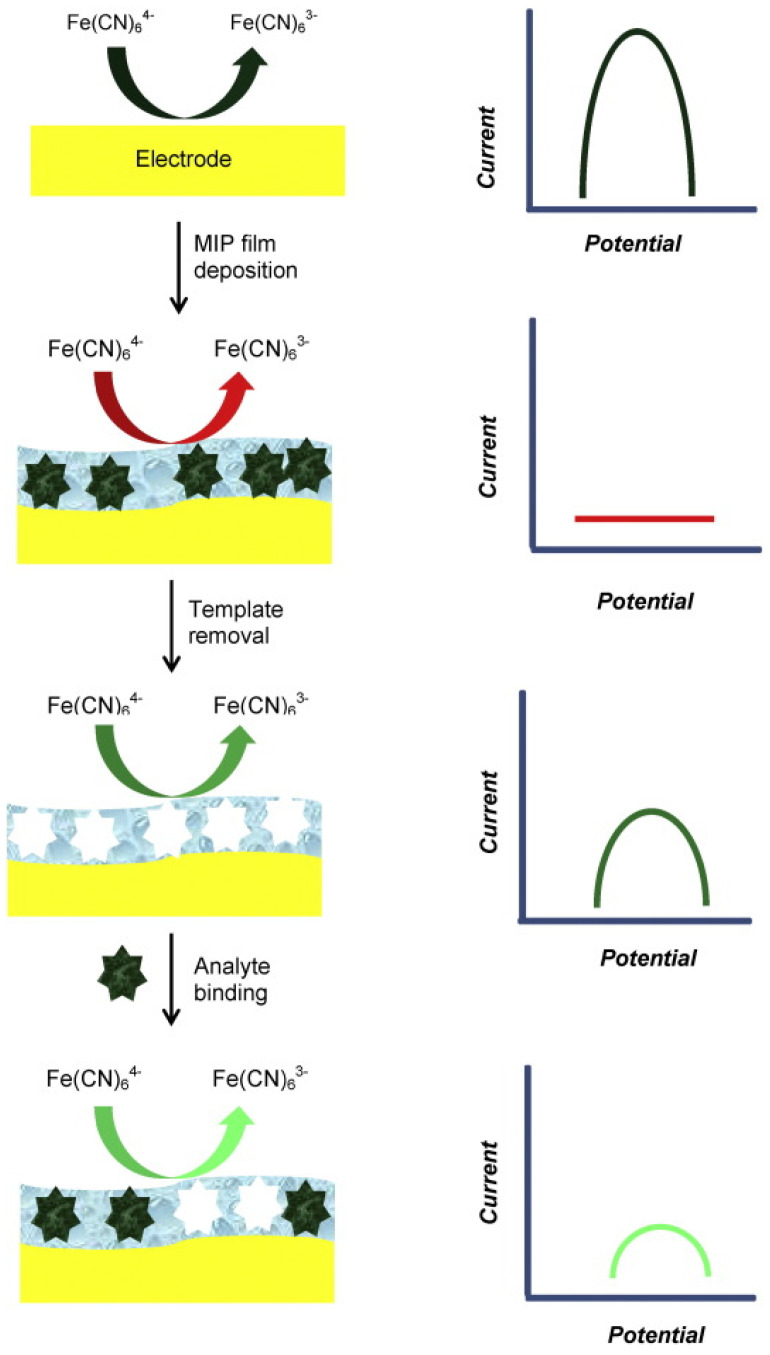
Examples of using “gating effect” for indirect electrochemical detection of the analyte at the MIP-based electrode and the corresponding current-potential signals. Reprinted with permission from [15]. Copyright 2016 Elsevier.

As mentioned above, few MIP-based electrochemical sensors provided electrochemical readout with direct oxidation or reduction of analytes. Alternatively, an indirect method using a redox probe is preferable. Here, a suitable redox probe’s electrochemical oxidation or reduction was observed in a test solution over a template-extracted and template-bound MIP film [167]. For the redox probe hexacyanoferrate, the cyclic voltammograms in the presence of a template molecule differed from those in its absence at the MIP-modified electrode (Figure 12) [7,167,168,169,170]. The mechanism, known as the “gate effect”, was first described by Yoshimi et al. [168] on an acrylic MIP-based chemosensor. Atomic force microscopy (AFM) images indicated that changes in the porosity of the MIP thin film during template binding were responsible for the sensitivity to the template. However, the exact recognition mechanism has not been fully elucidated [7,167,168].

When considering MIPs, the gate effect is primarily viewed as an electrode–electrolyte phenomenon influenced by the nature of the MIP film. In the “gate–key” analogy, the template acts as the “key”, and the molecular cavities in an MIP film represent the corresponding “keyhole”. The gating effect is created by affecting the faradaic process of a redox probe by filling the imprinted binding sites in a polymeric network with analyte molecules [167,168]. There are different types of interactions between MIP film and a template; therefore, different mechanisms can cause the gate effect [7,167].

### 5.1. Diffusion-Controlled Mechanism

#### 5.1.1. Shrinking and Swelling of MIP Film

It was first proposed that the binding of target analyte molecules by molecular imprints causes swelling of the MIP film, which leads to pore expansion in the polymer matrix [167,168,169]. Consequently, the diffusion of redox probe molecules through the film to the underlying electrode was facilitated. The increased diffusion rate significantly increased the faradaic current corresponding to the oxidation of the redox probe at the electrode–MIP film interface. It was later found that analyte binding can also suppress the intensity of the faradaic probe current. Such hindered or enhanced diffusion arises from the changes in the thickness and topography of the MIP-bound film [167,169,170].

#### 5.1.2. Physical Cavity Blocking by Analyte Molecules

According to this model, the impeded flow of redox probe molecules or ions through the MIP film may occur due to the physical blocking by the adsorbed analyte molecules [167,169]. For low-molecular-weight templates, the diameters of the imprinted cavities are often comparable to those of the redox probe [7,167], so the actual cause of diffusion is unclear. It is implausible that the redox probe molecules diffuse directly through the imprinted cavities. Moreover, it is unlikely that the cavities are fully interconnected to form long channels to reach the underlying electrode across the MIP film. Therefore, this mechanism can be attributed to macromolecular-imprinted thin MIP films. The presence of a non-conducting protein template molecule in its molecular cavity can block the diffusion of small redox probes. Such impairment of redox probe diffusion would manifest itself in a significant signal change of a redox probe [167,169].

### 5.2. Electronic Property-Controlled Mechanism

The gate effect mechanism controlled by the electronic properties of MIP postulates a change in MIP conductivity caused by analyte binding, which is considered to be a doping–“dedoping” effect [167]. For this reason, it can lead to both an increase and a decrease in the faradaic current of the redox probe. Generally, this mechanism affects eMIPs (i.e., MIPs prepared with electroactive functional monomers). It has been shown that the conducting MIP backbone carries delocalized π-electrons, whose mobility determines the conductivity of the polymer. In this view, the MIP backbone is enriched with repeated units compatible with energetically homogeneous electronic states. Defects can perturb the latter, due to the existence of modified units, such as non-conjugated bonds [167,169]. Therefore, there are at least two scenarios of events associated with the MIP recognition of the analyte molecule: (1) binding of the analyte into corresponding molecular imprints disrupts the electronic transport pathway, resulting in a low-conductivity state; (2) rebinding of the analyte enhances the electronic transport, characterized by increased conductivity. In addition, the changes in the MIP layer caused by analyte adsorption can lead to its passivation [167].

### 5.3. The Electrical Double Layer Model

The double-layer forms spontaneously at the electrode–electrolyte interface due to the interactions between the ions present in an electrolyte solution and the electrode surface. At the bare electrode, the electrical double layer formation depends on the electrolyte concentration. On the other hand, the double-layer formation is more complex at the MIP-based electrode. Alongside diffusion of ions or redox probe molecules, other processes are involved, including diffusion, accumulation, and selective adsorption of an analyte in an MIP. Consequently, the charge transfer and diffusion of the redox probe from the solution to the underlying electrode are affected by the character of the MIP film. The rebinding of the analyte into the molecular cavities can also affect the capacitance of the film itself [167].

The electrochemical properties of conducting MIP film depends on external conditions [167,171]. In the case of a neutral MIP film, it acts as an insulator or a semiconductor in its reduced form. After charging (by electrooxidation and counterion doping), it exhibits high conductivity, which may even be comparable to metals [167]. A prerequisite for the electro-oxidation of conductive polymers is the presence of counterions (i.e., dopants) that can neutralize the formation of the cation radicals (i.e., polarons) [167,169]. Electrooxidation or electroreduction of a conducting polymer is usually accompanied by its swelling or shrinkage. Therefore, it is unclear where the double layer is formed and where the electron transfer sites are located. There are two possibilities where the double layer can be located: (1) within an MIP film near the electrode surface; (2) at the polymer-solution interface, far from the underlying electrode [167].

## 6. Conclusions and Future Remarks

The development of biomimetic electrochemical sensors is a hot topic in electroanalysis. Incorporating MIPs—materials with antibody-like functions—into a sensing platform represents a new frontier for designing low-cost, sensitive, fast, and portable sensing devices. Compared to MIPs, which are fabricated using variants of the chemical approach, electrosynthesized MIPs have emerged as promising candidates for advanced sensors for a wide range of applications. Conventional biosensors for personalized medicine have not yet reached their full potential. However, technological advances have accelerated the development of e-health and (bio)sensing platforms. Several examples of MIP-based (bio)sensors have reached the required limit of detection and linear concentration range to be biologically/clinically relevant in recent years. Despite these analytical advantages, the main challenge of the MIP-based sensors remains the optimization of production methods that ensure the manufacture of sensor platforms that can detect biologically relevant values and enable mass production. However, with gradual technological development and the emergence of novel materials, MIP-based platforms are expected to become widely adopted. This review shows that MIPs can be compatible with smartphone readouts, making them suitable for commercial applications. Although it is currently difficult to produce MIPs in large batches that are homogeneous in size and shape without compromising the affinity to their target, the interest in expanding this promising technology will allow innovative solutions to overcome this hurdle. In addition, MIPs are very well suited to suppress interference in the electrochemical detection of low-molecular-weight targets by acting as a shape-selective sieve. The “gate effect” phenomenon of MIP-based electrochemical sensors has the disadvantage of producing an indirect signal, whose origin is still unclear. This reflects the presence of the target analyte and the changes associated with the target-polymer interactions. All in all, the continued development and generic nature of molecular imprinting technology promises the commercialization of MIP-based sensors that can be used as clinical point-of-care devices or as monitoring/diagnostic tools for home use.

## Figures and Tables

**Table 1:** Different synthetic routes for MIP formation.

Imprinting Method	Features
Bulk imprinting	The surface of the transducer is coated with a prepolymer mixture containing a small molecule template Before polymerization, the components interact with each other in a solution to form a network Entrapment of the template in the curing process Removal of the template with elution Addition of cross-linking agent
Surface imprinting	Support required for the imprinting process Imprinted binding sites are positioned close to the polymer surface Synthesis of thin polymer films More robust imprinting structure due to support Easy integration into electrochemical platforms
Microcontact imprinting	Stamp (usually a glass slide) is coated with a template by adsorption Prepolymers (monomers and cross-linkers) are introduced into the transducer support as an MIP substrate Polymerization occurs after contact between the template stamp and substrate Removal of the stamp and elution of the bound template Useful for the formation of highly sensitive, low cost and stable thin imprinted films
Polymer-brush imprinting	Based on the grafting of polymer chains onto a solid interface through one end Attachment of a template molecule to a polymer layer and the subsequent removal of the template molecule by enzymatic or chemical treatment A grafted polymer occupies the space in the vicinity of the adsorbed template The absorption of a template is often non-specific, resulting in poorer biosensor performance
Surface grafting	Useful for imprinting macromolecular structures (proteins, polysaccharides) or microorganisms Synthesis of a thin polymer film or use of a nanomaterial substrate as support for template attachment Imprinted binding sites are located near the surface of the outer polymer layer A limited number of binding sites Higher physical robustness Easy integration into electrochemical platforms
Epitope imprinting	For protein imprinting polymers, especially to target membrane proteins (recognition is by the part of the protein that is on the cell surface) Small, representative polypeptide sequences used as a template Very sensitive to any mismatch in the amino acid sequence (lower selectivity of detection)
Electropolymerization	Usually by applying a suitable potential or range of potentials to a solution containing the template and monomer Film formation on the surface of the electrode Close control by polymer thickness (adjusting electrochemical conditions, using different conductive materials) Usually, no cross-linker is required

**Table 2:** Summary of some eMIP-based sensors for clinically relevant biomarker, featuring polymer matrix, electrochemical detection method, linear concentration range and detection limit.

Target	MIP	Electrode Material	Detection Method	Linear Centration Range	LOD	Reference
gp51	Ppy	Pt	PDA (Photodiode-Array)	-	-	[107]
*B. subtilis* endospores	Ppy/poly (3-methylthiophene)	GCE	EIS (electrochemical impedance spectroscopy)	10^4^–10^6^ CFU/mL	10^2^ CFU/mL	[108]
EP	PAPBA	MWCNTs/GCE	DPV (differential pulse voltammetry)	0.2–800 µM	35 nM	[109]
Insulin	PPD	GCE	DPV	10^−14^–5 · 10^−13^ M	7.24 10^−15^ M	[71]
BuCHE	PPD	GCE	CV, amperometry	0.05–2 nM	14.7 pM	[110]
VEGF	PPD	SPE	EIS	20–200 pg/mL	0.08 pg/mL	[111]
ncovNP	PPD	Au-TFE	DPV	≤111 fM	15 fM	[112]
dopamine	poly(*o*-aminophenol)	Au	DPV	20–250 nM	1.98 nM	[113]
NE	poly(*o*-aminophenol)	GCE	SWV	0.05–10 µM	0.49 nM	[114]
CA15-3	poly(2-aminophenol)	AuSPE	DPV	5–50 U/mL	1.5 U/mL	[90]
HER2-ECD	polyphenol	AuSPE	DPV	10–70 ng/mL	1.6 ng/mL	[115]
3-NT	polyphenol	Paper-based with carbon ink	DPV	500 nM–1 mM	22.3 nM	[116]
cyt c	polyscopoletin	Au/MUA	CV, chronoamperometry	-	-	[117]

## Data Availability

The data presented in this study are available on request from the corresponding author.

## Abbreviations

3-APBA	3-aminophenyl boronic acid
3-NT	3-nitrotyrosine
AC	alternating current
ANI	aniline
APBA	aminophenyl boronic acid
AuSPE	gold screen-printed electrode
Au-TFE	gold thin film electrode
BSA	bovine serum albumin
BTC	butyrylthiocholine iodide
BuChE	butyrylcholinesterase
CA15-3	cancer antigen 15-3
CNT	carbon nanotube
CS	chitosan solution
CV	cyclic voltammetry
cyt *c*	cytochrome *c*
DET	direct electron transport
DNA	deoxyribonucleic acid
DPA	dipicolinic acid
DPV	differential pulse voltammetry
EGTA	ethylene glycol tetraacetic acid
EIS	electrochemical impedance spectroscopy
eMIP	electro synthesized molecularly imprinted polymer
EP	epinephrine
FTIR	Fourier transform infrared spectroscopy
GCE	glassy carbon electrode
GSPE	graphene screen-printed electrode
HER2-ECD	extracellular domain of human epidermal growth factor receptor 2
HTHP	hexameric tyrosine-coordinated heme protein
ITO	indium-tin-oxide
LBL	layer-by-layer
LOD	limit of detection
MIP	molecularly imprinted polymer
*m*PD	*m*-phenylenediamine
MUA	mercaptoundecanoic acid
MWCNT	multi-walled carbon nanotubes
ncovNP	SARS-CoV-2 nucleoprotein
NE	norepinephrine
NIP	non-molecular imprinted polymer
NP	nanoparticle
*o*-PD	*o-*phenylenediamine
OS	oxidative stress
PANI	polyaniline
PAPBA	polyaminophenylboronic acid
PBS	phosphate-buffered saline
*PmPD*	poly-*m*-phenylenediamine
POC	point-of-care
PPD	poly(*o-*phenylenediamine)
PPy	polypyrrole
Py	pyrrole
RAFT	reversible addition-fragmentation chain transfer
ROS	reactive oxygen species
SAM	self-assembly monolayer
SEM	scanning electron microscopy
SWV	square-wave voltammetry
VEGF	vascular endothelial growth factor
